# Abnormal leaf development of *rpt5a* mutant under zinc deficiency reveals important role of DNA damage alleviation for normal leaf development

**DOI:** 10.1038/s41598-019-44789-w

**Published:** 2019-06-27

**Authors:** Naoyuki Sotta, Takuya Sakamoto, Sachihiro Matsunaga, Toru Fujiwara

**Affiliations:** 1 Department of Applied Biological Chemistry, Graduate School of Agricultural and Life Sciences, The University of Tokyo, 1-1-1 Yayoi, Bunkyo Tokyo, 113-8657 Japan; 2 Department of Applied Biological Science, Faculty of Science and Technology, Tokyo University of Science, 2641 Yamazaki, Noda Chiba, 278-8510 Japan

**Keywords:** Leaf development, Abiotic

## Abstract

Leaf development in plants, including dorsoventral (adaxial–abaxial) patterning, is tightly regulated. The involvement of several subunits of the 26S proteasome in adaxial–abaxial polarity establishment has been reported. In the present study, we revealed that in *Arabidopsis thaliana*, a mutation in *RPT5A*, a subunit of 26S proteasome, causes abnormally narrow true leaves under zinc deficiency. mRNA accumulations of DNA damage marker genes in leaves were elevated by zinc deficiency. *PARP2*, a single-strand break (SSB) inducible gene, was more strongly induced by zinc deficiency in *rpt5a* mutants compared with the wild type. A comet assay indicated that SSB is enhanced in mutants grown under the zinc deficiency condition. These results suggest that SSB accumulation is accompanied by abnormal leaf development. To test if DNA damage is a sole cause of abnormal leaf development, we treated the wild type grown under normal zinc conditions with zeocin, a DNA damage-inducing reagent, and found that narrow leaves developed, suggesting that DNA damage is sufficient to induce the development of abnormally narrow leaves. Taken together with the observation of the abnormal leaf morphology of our mutant plant under zinc deficiency, we demonstrated that the alleviation of DNA damage is important for normal leaf development.

## Introduction

In angiosperms, the shoot apical meristem (SAM) generates leaves by differentiating a part of it as leaf primordia. Flat leaf formation requires the establishment of two positional axes in positional relation to the SAM: the proximal-distal axis and the adaxial–abaxial axis. Adaxial–abaxial polarity determination is controlled by multiple families of transcription factors^1^. *PHABULOSA* (*PHB*), *PHAVOLUTA* (*PHV*), and *REVOLUTA* (*REV*), all class III HOMEODOMAIN-LEUCINE ZIPPER (HD-ZIPIII) genes, are known as adaxial fate contributors. Dominant mutations in *PHB* and *PHV* cause a dramatic transformation of abaxial leaf fates into adaxial leaf fates^2,3^. *ASYMMETRIC LEAVES* (*AS*) 1 and *AS2* form a complex and are required to establish adaxial cell fate^4^. The constitutive expression of *AS2* results in the adaxialized phenotype^4^. *KANADI* (*KAN*) and *YABBY* (*YAB*) genes specify abaxial fate in cells, being expressed in the abaxial face^5–7^. Leaf axes are established by a complex regulatory network including these transcription factors as well as signaling through miRNA and hormone signaling^8^. The plant developmental process in general is affected by environmental conditions, but to our knowledge, the conditional determination of adaxial–abaxial polarity has not been reported.

It has been demonstrated that the protein degradation pathway mediated by the 26S proteasome participates in leaf adaxial–abaxial polarity formation and that this pathway acts in parallel with other pathways, including the *AS1-AS2* transcriptional factor pathway^9^. Huang *et al*. demonstrated that defects in the 26S proteasome enhance the *as2* abnormal leaf phenotype. They showed that several proteasome subunits are essential for specifying leaf adaxial identity and that a mutation in *RPN8A*, a regulatory particle subunit, increases transcript levels of abaxial-promoting genes and results in very narrow blade leaves with a phloem-surrounding-xylem structure or radically symmetric needle-like leaves with no apparent vascular bundle. The disruption of other regulatory particles has also been reported to have a pleiotropic effect on leaf development. Mutants of *RPT2A* exhibit enlarged rosette leaves with enlarged cell size, which has been attributed to extended endoreduplication^10^. *RPN10* is suggested to be involved in abscisic acid signaling, and its mutant exhibits a pleiotropic phenotype with a slower rate of leaf initiation and expansion and premature senescence^11^. *RPN12* is considered to be involved in cytokinin regulation, and *rpt12a-1* exhibits a slower rate of rosette leaf emergence^12^.

In the present work, we revealed that defects of a 26S proteasome subunit *RPT5A* result in abnormal, filamentous leaf formation under the zinc deficiency condition without *as1* or *as2* mutations. The 26S proteasome RPT5 subunit is encoded by two paralogous genes: *RPT5A* and *RPT5B*. In Col-0 accession, it is known that *RPT5B* compensates *rpt5a* mutation to some extent, but the functions of the two are not identical^13^. Previously, we reported that *rpt5a-4* has reduced proteasome activity and increased accumulation of poly-ubiquitinated proteins^14^. It revealed that *rpt5a-4* exhibits reduced shoot growth under zinc deficiency but not under normal conditions, suggesting that *RPT5A*, but not *RPT5B*, is involved in zinc deficiency tolerance, possibly through the alleviation of oxidative stress and/or processing of polyubiquitinated proteins. Zinc is known as a cofactor for Cu/Zn-superoxide dismutase (SOD), a major reactive oxygen species scavenger, and its deficiency decreases SOD activity in plants^15,16^. However, the relationship between the increased oxidative stress and leaf morphology remains unknown.

The possible involvement of oxidative stress in abnormal leaf development motivated us to examine the involvement of DNA damage, because generally, oxidative stress induces DNA damage^17^. The effects of the DNA damage response on leaf development have been reported. One effect is in cell compensation, where reduced cell division is compensated by increasing cell size to maintain the final leaf area (reviewed by^18^). An analysis of the *fas1* (*fasciata1*) mutant, which suffers from endogenous DNA damage, suggested that the *ATAXIA TELANGIECTASIA MUTATED* (*ATM*)-dependent DNA damage response is a trigger for compensated cell expansion^19^. Another effect of DNA damage on leaf development has been found in the Elongator complex, an evolutionarily conserved histone acetyltransferase complex that is an important regulator of the mitotic cell cycle to promote leaf patterning^20^. Loss of Elongator functions induced aberrant DNA replication and increased DNA damage, resulting in severe leaf polarity aberrance^20^.

Through this work, we dissect the mechanism of abnormal leaf development under zinc deficiency to shed light on the effect of DNA damage on leaf development. By analyzing the *Arabidopsis thaliana rpt5a-4* mutant, we demonstrate that *RPT5A* is important for alleviating DNA damage under zinc deficiency and that DNA damage is sufficient to trigger the development of aberrant filamentous leaves.

## Results

### Zinc deficiency induced filamentous true leaves in *rpt5a-4* and *rpt2a-2* mutants

**Figure 1:**
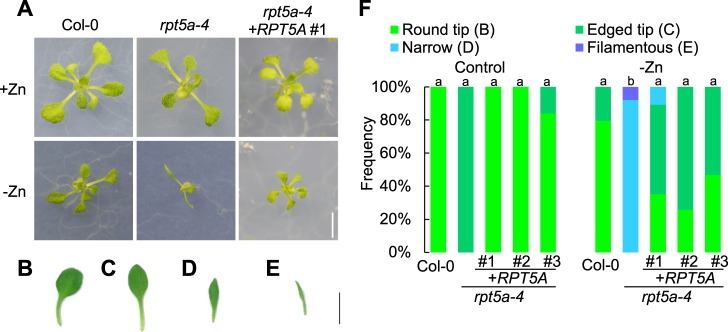
Leaf shape of *rpt5a-4* under zinc deficiency. (**A**) Fourteen-day-old seedlings of wild type, *rpt5a-4*, and *rpt5a-4* complementation line grown under control and zinc deficiency conditions. Bar; 5 mm. (**B–E**) Leaf shapes were categorized into four groups, round tip (**B**), edged tip (**C**), narrow (**D**), and filamentous (**E**), as illustrated in (**B**) from left to right. Bar; 5 mm. (**F**) Frequency was calculated from observation of at least 20 individual plants for each condition. The numbers with “#” are identifiers for independent transformant lines in the complementation experiment. There is no significant difference in the ratio of (“round tip” + “edged tip”): (“narrow” + “filamentous”) at *p* < 0.05 among groups sharing the same letter by Fisher’s exact test with Holm correction.

To investigate the involvement of *RPT5A* in leaf development under zinc deficiency, we observed first true leaves of the wild type and *rpt5a-4* grown under control (1 µM zinc) or zinc deficiency (no zinc added) conditions. Grown under the control condition, *rpt5a-4* true leaves exhibited morphological differences from the wild type (Fig. 1A). The wild type exhibited broader leaf blades and round leaf tips (Fig. 1B), while *rpt5a-4* exhibited narrower leaf blades and edged leaf tips (Fig. 1C). This phenomenon has also been mentioned previously^9^. Under the zinc deficiency condition, no significant effect on leaf morphology was observed in the wild type. On the other hand, *rpt5a-4* leaf morphology was significantly affected by zinc deficiency. The majority of leaves exhibited reduced leaf blade width, with an ambiguous boundary between leaf blades and petioles (Fig. 1D,F). Approximately 10% of true leaves exhibited more severe morphological alteration: their leaf blades were undeveloped and indistinguishable from petioles (Fig. 1E).

To genetically establish that the loss of function in *RPT5A* is responsible for the abnormal leaf formation under zinc deficiency, we conducted a complementation test by introducing a genomic fragment of *RPT5A* cloned from the wild type into *rpt5a-4*. We obtained three independent homozygous transformant lines. Expression of the transgene in these transformants was confirmed by qRT-PCR (Supplementary Fig. 1). All of the obtained transformant lines exhibited non- or less-frequent narrow or filamentous leaves compared with *rpt5a-4* under zinc deficiency conditions. This result indicates that introducing a *RPT5A* genomic fragment into *rpt5a-4* suppresses abnormal leaf formation in *rpt5a-4* under zinc deficiency, establishing that *RPT5A* is responsible for circumventing abnormal leaf formation under zinc deficiency.

To examine whether the observed phenomenon is specific to *RPT5A* among proteasome components, we observed leaf morphology of a mutant of another proteasome subunit, *RPT2A*. As is the case in *rpt5a-4*, *rpt2a-2* exhibited narrow leaf blades or filamentous leaves under zinc deficiency (Supplementary Fig. 2), suggesting that involvement in leaf development under zinc deficiency is not specific to *RPT5A*, but at least common between the two proteasome components.

### Adaxial identity was defective in *rpt5a-4* leaves under zinc deficiency

A needle-like leaf structure is generally thought to be a result of defective leaf adaxial–abaxial polarity^9^. To investigate the alteration in adaxial–abaxial fate, we observed trichomes and vascular bundle patterns in the blade–petiole conjugation region in *rpt5a-4* under zinc deficiency.

**Figure 2:**
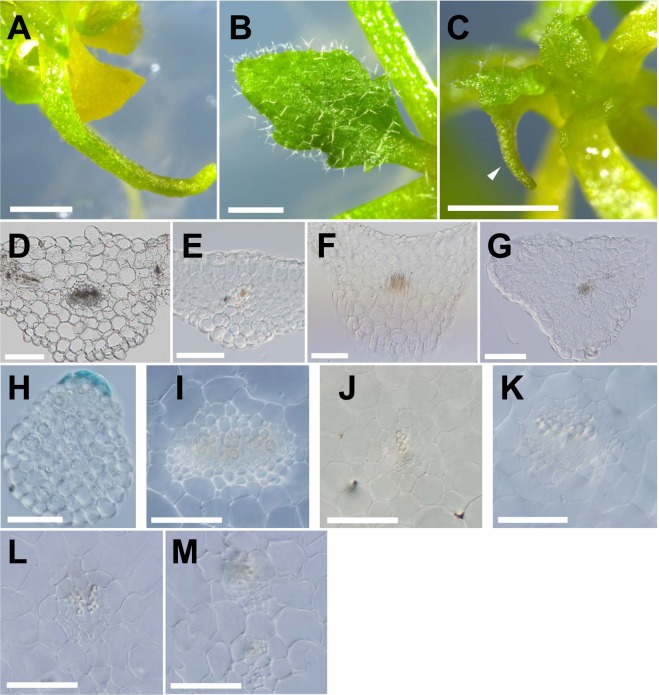
Aberrant leaf development in *rpt5a-4* leaves under zinc deficiency. (**A**) Filamentous true leaf of *rpt5a-4* under zinc deficiency. Note that no trichome has been developed. (**B**) *rpt5a-4* leaf under control (1 µM zinc) condition (**C**) SAM of *rpt5a-4* under zinc deficiency. Arrowhead indicates abnormal horn-like structure developed from emerging leaf. (**D**,**E**) Cross-sections of leaves of wild type under control (**D**) and zinc deficiency (**E**) conditions. (**F**,**G**) Cross-sections of leaves of *rpt5a-4* under control (**F**) and zinc deficiency (**G**) conditions. (**H**) Cross-section of most severely filamentous leaf of *rpt5a-4* under zinc deficiency. Blue stain is marker ink put on adaxial leaf surface before slicing. (**I**,**J**) Vascular bundle of wild type under control (**I**) and zinc deficiency (**J**) conditions. (**K**–**M**) Vascular bundle of *rpt5a-4* under control conditions (**K**) and filamentous leaves under zinc deficiency (**L**,**M**). Bars, (**A**,**B**) 1 mm; (**C**) 500 µm; (**D**–**H**) 100 µm; (**I**–**M**) 50 µm.

No trichome development was observed in severely filamentous leaves of *rpt5a-4* under zinc deficiency, whereas *rpt5a-4* under the control condition developed trichomes on the adaxial leaf surface (Fig. 2A,B), suggesting that adaxial identity was not properly established in *rpt5a-4* under zinc deficiency. Under zinc deficiency, *rpt5a-4* infrequently developed a horn-like structure from the abaxial side of emerging leaves (Fig. 2C). The horn-like structure was not consecutive to the filamentous leaf development. Observation of the cross-sections revealed that filamentous *rpt5a-4* leaves under zinc deficiency did not develop the expanded leaf blade but had the midrib alone, whereas the *rpt5a-4* under control conditions and the wild type under either condition developed the leaf blade (Fig. 2D–G). The most severely filamentous leaves did not develop the leaf blade at all (Fig. 2H).

In the wild-type vascular bundle, xylem is developed in the adaxial side, and phloem is developed in the abaxial side (Fig. 2I,J). The relative positioning of xylem and phloem was maintained in filamentous *rpt5a-4* leaves (Fig. 2K–M). However, the vascular bundle and tracheary element of xylem were thinner in *rpt5a-4*, which was more evident under zinc deficiency. In some filamentous leaves, two independent vascular bundles were observed (Fig. 2M). The most severely filamentous leaves did not develop the vascular bundle (Fig. 2H). These observations suggest that adaxial identity is defective in *rpt5a-4* leaves developed under zinc deficiency.

### Expression of abaxial determinant genes was suppressed in *rpt5a-4* leaves under zinc deficiency

**Figure 3:**
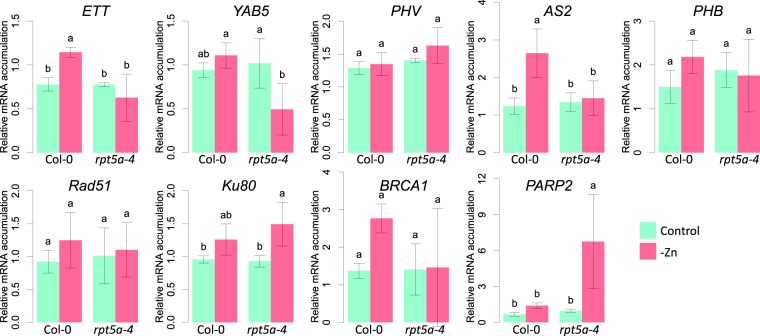
mRNA accumulation in *rpt5a-4* mutant under zinc deficiency. Seedlings were grown for 14 days and total RNA was extracted from the first pair of true leaves with the representative shapes; for the control condition, the wild type and *rpt5a-4* were represented by “round tip” and “edged tip”, respectively, and for the zinc deficiency condition, the wild type was represented by “round tip” and “edged tip,” whereas *rpt5a-4* was represented by “narrow” or “filamentous.” mRNA accumulations were normalized by the geometric mean of *Actin8*, *PEX4*, and *SAND*. Values represent mean ± standard deviation of four biological replicates. Groups sharing the same letter were not significantly different from each other at *p* < 0.05 by Tukey’s multiple test.

To investigate possible effects of zinc deficiency on leaf development, we examined the expression of adaxial and abaxial marker genes in the developing true leaves (Fig. 3). RNA was extracted from round-tip leaves from the wild type under the control or zinc deficiency conditions, edged-tip leaves from *rpt5a-4* under the control condition, and narrow or filamentous leaves from *rpt5a-4* under the zinc deficiency condition. qRT-PCR revealed that mRNA accumulation of the adaxial marker gene, *AS2*, was reduced, whereas *PHV* and *PHB* were not significantly affected by zinc deficiency or by *rpt5a-4* mutation. On the other hand, abaxial marker genes, *ETTIN* (*ETT*) and *YAB5*, were significantly reduced in *rpt5a-4* under zinc deficiency. These results suggest that *RPT5A* is required for the maintenance of adaxial–abaxial balance under zinc deficiency.

### DNA damage was accumulated in *rpt5a-4* leaves under zinc deficiency

To investigate the possible involvement of DNA damage in leaf development under zinc deficiency, we first measured mRNA accumulation of four DNA damage marker genes in shoots by qRT-PCR (Fig. 3). *Rad51*, *Ku80*, and *BRCA1* tended to be induced by zinc deficiency, but no significant differences were detected between those of the wild type and *rpt5a-4*. Among the four genes, only *PARP2* (At4g02390) exhibited a difference between the wild type and *rpt5a-4* under zinc deficiency. In *PARP2*, induction by zinc deficiency was evident in *rpt5a-4* but not significant in the wild type.

**Figure 4:**
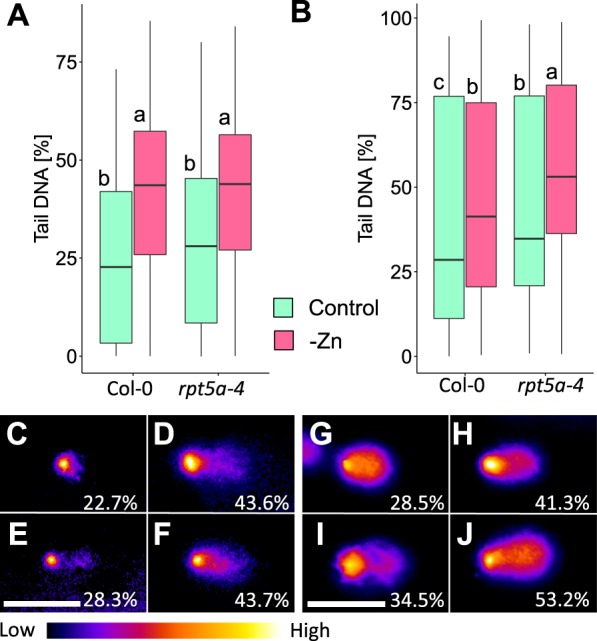
DSB and SSB accumulations in shoot of *rpt5a-4* under zinc deficiency. Nuclei were extracted from whole shoots of 16-day-old seedlings and were subjected to a comet assay. (**A**) N/N comet assay to detect DSB. (**B**) A/N comet assay to detect DSB and SSB. At least 100 nuclei were observed for each treatment. Median and first and third quartiles are shown in cross bars and vertical bars represent range of data points obtained. There is no significant difference at *p* < 0.05 between groups sharing the same letter by Steel–Dwass test. (**C**–**J**) Representative nuclei images from comet assay with N/N method (**C**–**F**) and A/N method (**G**–**J**). Nuclei with median Tail DNA [%] are shown with their values. (**C**,**G**) Wild type, control, (**D**,**H**) wild type, zinc deficiency, (**E**,**I**) *rpt5a-4*, control, (**F**,**J**) *rpt5a-4*, zinc deficiency. Bars, 50 µm. Contrast and brightness were adjusted for visualization purposes. A pseudo-color scale is shown at the bottom.

To assess DNA damage accumulation directly, we conducted a comet assay. We adopted two variations of the comet assay to distinguish a double-strand break (DSB) from a single-strand break (SSB): the neutral/neutral (N/N) method detects mainly DSB, while the alkali/neutral (A/N) method detects DSB and SSB^21^. Tail DNA percentage detected by the N/N method was increased by zinc deficiency in both genotypes, but no significant differences were detected between the wild type and *rpt5a-4* (Fig. 4A). Tail DNA percentage detected by the A/N method was induced by zinc deficiency, and the levels were higher in *rpt5a-4* compared with those of the wild type (Fig. 4B). These results suggest that zinc deficiency causes DNA damage in shoots and that *RPT5A* is involved in the alleviation of SSB in shoots.

### DNA damage was sufficient to induce development of filamentous leaves

**Figure 5:**
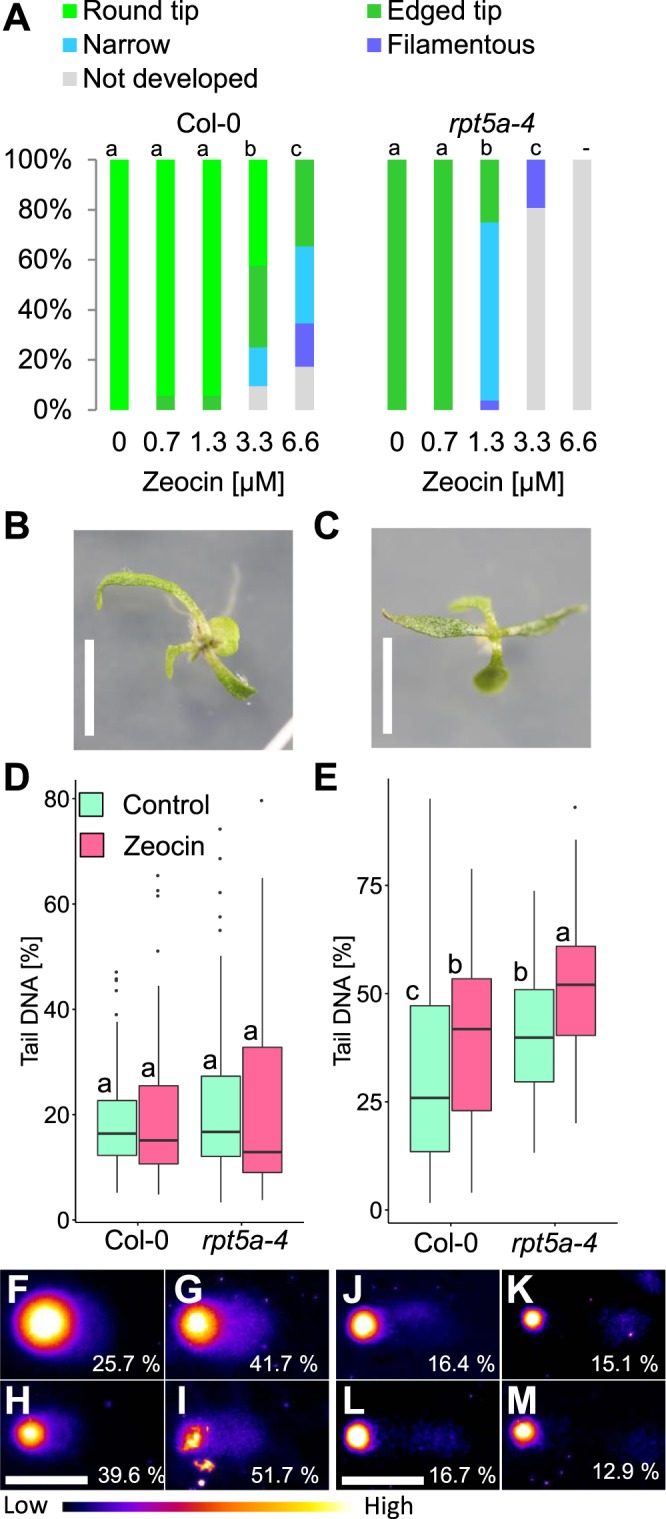
DNA damage-inducing treatment caused filamentous leaves in a dose-dependent manner. Seedlings were grown under treatment with various concentrations of zeocin, a DNA damage-inducing reagent. Zeocin was added to the normal medium (MGRL medium with normal zinc). (**A**) Frequency of aberrant leaves observed under treatment with various concentrations of zeocin. For each condition, 52 seedlings were observed. There is no significant difference in the ratio of (“round tip” + “edged tip”): (“narrow” + “filamentous”) at *p* < 0.05 among groups sharing the same letter by Fisher’s exact test with Holm correction. (**B**) Seventeen-day-old wild type under 6.6 µM zeocin treatment. (**C**) *rpt5a-4* under 3.3 µM zeocin treatment. Bars, 5 mm. (**D**,**E**) DNA damage accumulations in shoot of *rpt5a-4* grown under 3.3 µM zeocin treatment: (**D**) the N/N comet assay to detect double strand break, (**E**) the A/N comet assay to detect double and single strand break. Nuclei were extracted from whole shoots of 14-day-old seedlings and were subjected to comet assay. At least 100 nuclei were observed for each treatment. There is no significant difference at *p* < 0.05 between groups sharing the same alphabets by Steel-Dwass’ test. (**F**–**M**) Representative nuclei images from the comet assay with N/N method (**F–I**) or A/N method (**J–M)**. Nuclei which give median Tail DNA [%] are shown with its value. (**F**,**J**) Wild type, control, (**G**,**K**) wild type, 3.3 µM zeocin, (**H**,**L**) *rpt5a-4*, control, (**I**,**M**) *rpt5a-4*, 3.3 µM zeocin. Bar, 50 µm. Contrast and brightness were adjusted for visualization purpose. Pseudo-color scale is shown in the bottom.

As DNA damage was highly accumulated in *rpt5a-4* shoots under zinc deficiency, we hypothesized that the abnormal leaf morphology results from the accumulation of DNA damage. To test this hypothesis, we treated the seedlings with zeocin, a DNA damage-inducing reagent, to artificially induce DNA damage, and investigated whether it affected leaf morphology. In *rpt5a-4*, zeocin treatment with a concentration range of 1.3–3.3 µM produced filamentous or narrow leaves similar to those observed under zinc deficiency (Fig. 5A,C). With the severer treatment with 6.6 µM zeocin, *rpt5a-4* was not viable, whereas the wild type was able to develop true leaves, suggesting that *rpt5a-4* is more sensitive to DNA damage. The induction of filamentous or narrow leaves by zeocin was observed even in the wild type, although the sensitive dose range was higher than that of *rpt5a-4*, ranging from 3.3–6.6 µM (Fig. 5A,B).

To test whether the observed difference between the wild type and *rpt5a-4* in sensitivity to zeocin treatment was attributable to a difference in DNA damage repair ability, we conducted a comet assay with *rpt5a-4* treated with 3.3 µM zeocin (Fig. 5D–M). With A/N method, which detects SSB and DSB, higher damage accumulation was observed in *rpt5a-4* treated with zeocin (Fig. 5D), whereas no significant difference was detected with N/N method, which detects DSB alone (Fig. 5E). This suggests that it is SSB rather than DSB that is highly accumulated in *rpt5a-4* under zeocin treatment.

To test the generality of this DNA damage-induced abnormal leaf development in other types of abiotic stress, we observed the leaf morphology of *rpt5a-4* under excess boron stress, which is known to induce DNA damage^22^. The wild type developed normal leaf morphology under toxic conditions with 3 mM boric acid (Supplementary Fig. 3). On the other hand, with 3 mM boric acid, most *rpt5a-4* true leaves had reduced leaf blade width, with an ambiguous boundary between leaf blades and petioles (Supplementary Fig. 3 B). Comet assay revealed that excess boron stress resulted in DNA damage accumulation pattern which is similar to zeocin treatment (Supplementary Fig. 3C–L). With A/N method, higher damage accumulation was observed in *rpt5a-4* under 3 mM boric acid (Supplementary Fig. 3D), whereas no significant difference was detected with N/N method (Supplementary Fig. 3C), indicating that excess boron stress induces SSB in shoots. These results suggest that the mechanism of developing abnormal leaves where *RPT5A* plays a crucial role is not specific to zinc deficiency conditions but also occurs under abiotic stress conditions where DNA damage accumulation is involved, at least with excess boron stress.

## Discussion

In this work, we demonstrated that proteasome components, at least *RPT5A* and *RPT2A* are essential for normal leaf development, especially under a specific nutritional environment. The combination of *rpt5a-4* mutation and zinc deficiency or excess boron triggers severely aberrant leaves, but each factor alone does not (Figs 1 and S3). The phenotype of *rpt5a-4* leaves under zinc deficiency or excess boron conditions is similar to that of the *ae3-1* mutant: narrow or needle-like rosette leaves, which are attributed to a defective adaxial identity^9^. The *ae3-1* mutation was isolated as an enhancer of *as2-101* and identified as a mutation in *RPN8a*, a lid subunit of the 26S proteasome^9^. Huang *et al*. reported that abnormal rosette leaves were also observed in a selfed F_2_ population of a cross between *as2-101* and other proteasome subunit mutants: *rpt2a*, *rpt4a*, *rpn1a*, *rpn8a*, *rpn9a*, *pad1*, *pbe1*, as well as *rpt5a*. Based on this result, they suggested that the 26S holoenzyme plays a role in regulating leaf patterning. Along this line, our findings can be interpreted as indicating a zinc deficiency or excess boron stress effect on *rpt5a-4* phenocopying aberrant leaf formation in double mutants of *as2* and proteasome mutants, which suggests that these stress conditions affect the *AS2* pathway in leaf adaxial–abaxial determination.

Morphological observation revealed that adaxial–abaxial determination failed in *rpt5a-4* under zinc deficiency. The observations, including the absence of trichomes, absence of an expanding leaf blade, and reduction in *AS2* expression, suggest that the adaxial identity was defective. In addition, abaxial determinant genes, *ETT* and *YAB5*, were less accumulated in *rpt5a-4* than in the wild type. Given that the expression of both abaxial and adaxial marker genes is reduced in *rpt5a-4* under zinc deficiency, it can be interpreted to indicate that the abaxial–adaxial determination fails and the tissue is undifferentiated.

Our results demonstrated that DNA damage-inducing treatment was sufficient to induce abnormal leaf development even in the wild-type plants. In this context, considering that DNA damage accumulated more in *rpt5a-4* than in the wild type under zinc deficiency (Fig. 4), it is deduced that *RPT5A* contributes to normal leaf development through the alleviation of DNA damage induced by zinc deficiency. This is supported by the results that zeocin treatment induced abnormal leaf formation in both the wild type and *rpt5a-4* and that the sensitivity was higher in *rpt5a-4* than in the wild type. How does *RPT5A* alleviate DNA damage? Under zinc deficiency, the level of lipid peroxidation, an indicator of oxidative stress level, is higher in *rpt5a-4* and *rpt2a-2* compared with the wild type^14^. This suggests that these proteasome subunits are involved in alleviating oxidative stress. As oxidative stress induces DNA damage^17^, it is deduced that the reduced capacity for alleviating oxidative stress in *rpt5a-4* resulted in DNA damage accumulation. We do not exclude the possibility that *RPT5A* is also involved in other pathways, namely DNA damage prevention and/or repair.

Zinc deficiency tended to induce the expression of the four DNA damage marker genes we examined (Fig. 3). However, a significant difference between the wild type and *rpt5a-4* under zinc deficiency was detected only in *PARP2*. In mammalian cells, it is described that *PARP1* and *PARP2* contribute to SSB repair/base excision repair processes^23^. The direct measurements of DSB and SSB suggest that zinc deficiency induces both DSB and SSB and *RPT5A* is involved in the alleviation of SSB under these conditions (Fig. 4). Besides the DNA damage response, the induction of *PARP* in *rpt5a-4* under zinc deficiency itself may be involved in leaf development. *PARP* is known to be involved in the stress response and growth control of plants^24,25^. Schulz *et al*. found that PARP activity is involved in leaf cell number by demonstrating that the inhibition of PARP activity by 3-Methoxybenzamide (3MB) treatment results in an increased cell number with more cell divisions, especially in young leaves. In light of this, the increased expression of *PARP2* may result in reduced cell division in developing leaves, which could affect the leaf morphology.

Since *rpt5a-4* mutation affected SSB, but not DSB, we speculate that it is SSB rather than DSB that induces abnormal leaf formation. From this point of view, the notion that a well-known DSB-causing reagent, zeocin, induced abnormal leaf development might sound controversial. However, although zeocin is generally regarded as a DSB-inducing reagent, it is reasonable to believe that it also induces SSB, because a structurally related DNA damage-inducing reagent, bleomycin, is expected to react with oxygen to generate free radicals, resulting in SSB as well as DSB^26–28^. Although zinc deficiency but not excess boron or zeocin treatment induced DNA damage which is detected by N/N method comet assay, with A/N method we observed high damage accumulation in *rpt5a-4* consistently among these three treatments (Figs 4 and 5D–E, Supplementary Fig. 3C–D). These results also support the idea that SSB rather than DSB is responsible for the abnormal leaf formation.

Our finding is not the first instance of the involvement of DNA damage in leaf development in a broad sense. An analysis of *fas1*, a mutant of a large subunit of chromatin assembly factor-1 (CAF-1), revealed that DNA damage induces endoreduplication and increases leaf cell size^29^. However, in the present work, we demonstrated that DNA damage is involved in not only cell size determination but also developmental axis determination. We believe that our findings introduce a new perspective of leaf development. Intensive research studies have revealed the complex regulatory network of leaf generation from the SAM, including transcriptional factors, siRNA, and hormonal regulations, which have been considered as programmed development (review by^1,8^). However, the involvement of DNA damage, which is closely related to abiotic stress, in developmental axis determination implies the possible intervention of environmental conditions even in rather early stages of post-embryonic development.

## Materials and Methods

### Plant materials and growth conditions

As the wild type, ecotype Col-0 was used. Isolation of T-DNA knock out mutant *rpt5a-4* (SALK_046321) and *rpt2a-2* (SALK 005596) is described previously^14^.

Sterilized seeds were germinated and grown on MGRL medium^30^ supplemented with 1% w/v sucrose (BioUltra grade, Sigma-Aldrich, St. Louis, MO, USA), solidified with 1% w/v agar purified (Nacalai Tesque, Kyoto, Japan). For zeocin treatment, Zeocin Selection Reagent (Thermo Fisher Scientific, Yokohama, Japan) was added at the indicated concentrations. Medium plates were horizontally placed in a growth chamber (22 °C 16-h light/8-h dark cycle).

### Generation of transgenic plants

To generate *rpt5a-4* complementation lines, a genomic fragment of *RPT5A* containing 1062 bp upstream and 1040 bp downstream was cloned from Col-0 genomic DNA with primers RPT5A_F and RPT5A_R (Supplementary Table 2). The fragment was cloned into pENTR-D/TOPO following the manufacturer’s protocol (Invitrogen, Carlsbad, CA, USA) and then subcloned into a Gateway destination vector, pMDC99^31^, by LR recombination with LR Clonase II following the manufacturer’s protocol (Invitrogen). The constructed plasmid was mobilized into *Agrobacterium tumefaciens* (GV3101 pMP90) for transformation by the floral dip method^32^. Transgenic plants were selected on medium containing 1/2 × Murashige and Skoog, 1% sucrose, 20 μg/ml Hygromycin B, and 250 μg/ml Claforan. T4 transgenic plants harboring T-DNA homozygously were used for the analyses. To confirm the expression of the transgene, two to three pairs of the developing second or third true leaves were collected from 1-month-old seedlings. Total RNA was prepared using an RNeasy Plant Mini Kit (Qiagen, Tokyo, Japan) following the manufacturer’s protocol. Approximately 1 μg of total RNA was reverse-transcribed with a Verso cDNA Synthesis Kit (Thermo Fisher Scientific, Yokohama, Japan) following the manufacturer’s protocol. The resultant cDNA was used as a real-time PCR template with 10 times dilution. Real-time PCR was performed with Thermal Cycler Dice Real Time System II (Takara Bio, Shiga, Japan) and Luna qPCR Master Mix (New England Biolabs, Tokyo, Japan) using the primers listed in Supplementary Table 1.

### mRNA quantification by qRT-PCR

For qRT-PCR, 5–15 pairs of the developing first true leaves were collected from 14-day-old seedlings depending on the sample mass. Total RNA was prepared using NucleoSpin RNA Plant (MACHEREY-NAGEL, Düren, Germany) following the manufacturer’s protocol. About 500 ng of total RNA was reverse-transcribed with PrimeScript RT Master Mix (Perfect Real Time) (Takara Bio) following the manufacturer’s protocol. The resultant cDNA was used as a real-time PCR template with 3 to 10 times dilution. Real-time PCR was performed with Thermal Cycler Dice Real Time System II (Takara Bio) and SYBR Premix Ex Taq II (Tli RNaseH Plus) (Takara Bio) using the primers listed in Supplementary Table 1. For normalization among samples, the normalization factor was calculated by taking the geometric mean of three genes: *ACTIN8*, *PEX4*, and *SAND*^33,34^. The qualitative reproducibility of the results was confirmed with two independent experiments with three or four biological replicates.

### DNA damage assessment by comet assay

For nuclear isolation, shoots of 5 to 20 seedlings were minced in PBS with a razor blade approximately 100 times, followed by filtration through a CellTrics 30-µm filter (Sysmex Partec, Görlitz, Germany) with a centrifuge at 800 *g* for 1 min. For the neutral/neutral (N/N) method^21^, 10 µL of the supernatant was taken from the bottom of the tube. For the alkali/neutral (A/N) method^21^, the chloroplast was removed by repeating the following steps: 1. Add 10% v/v of 10× suc. sln. (50% sucrose, 2% Triton-X100 in PBS) to the supernatant and mix it well by inversion. 2. Centrifuge 1 min at 8,000 *g* and remove the supernatant. 3. Resolve the pellet in 450 µL of PBS. Steps 1 to 3 were repeated to obtain a white pellet. In Step 3 of the final iteration, the pellet containing nuclei was suspended in 20 µL of PBS.

The comet assay was performed using a CometAssay Kit (25 × 2 well slides) (Trevigen, Gaithersburg, MD, USA) with the following modifications. First, 10 µL of the nuclear extract was combined with 100 µL molten LMAgarose held at 37 °C, and 50 µL of the mixture was immediately pipetted onto CometSlide. The slides were kept at 4 °C in the dark for 30 min. The slides were immersed in a lysis solution (2.5 M NaCl, 10 mM Tris-HCl, pH8.0, 100 mM EDTA (self-prepared, not the one supplied in the kit)) (for the N/N method) or alkaline unwinding solution (0.3 M NaOH, 5 mM EDTA) (for the A/N method) for 20 min at room temperature. The slides were washed three times with TBE buffer for 5 min on ice. The slides were subjected to electrophoresis on ice with TBE buffer 1 V/cm for 6 and 4 min for the N/N method and the A/N method, respectively. The slides were soaked in 1% Triton-X100 in PBS for 10 min at room temperature and then washed twice with 70% ethanol for 5 min and twice with 96% ethanol for 5 min. The slides were dried at 37 °C for 1 h. For staining the nuclei, 100 µL of SYBR Gold staining solution (SYBR Gold Nucleic Acid Gel Stain (10,000 × Concentrate in DMSO, Thermo Fisher Scientific) diluted 30,000 times with TE pH 8.0) was placed onto CometSlide and kept for 30 min at room temperature in the dark. The slices were rinsed in water and dried completely at 37 °C. The fluorescence was observed with fluorescent microscope BX50WI with filter unit U-MWIBA3 (OLYMPUS, Tokyo, Japan), and the images were captured with an equipped CCD camera (OLYMPUS).

Images were quantified with CASP^35^ and with ImageJ macro OpenComet^36^ for the N/N method and the A/N method, respectively.

### Histological observation

For the observation of leaf cross-sections, fresh leaves were mounted in 45 °C molten agar (gelling temperature 30–31 °C, Nacalai Tesque) and solidified at 4 °C for 3 h. After being trimmed, gel blocks containing leaves were sliced into ~80-µm-thick slices by a vibrating microtome (Micro Slicer Zero 1; Dosaka EM, Osaka, Japan). Leaf slices were collected onto a glass plate for microscopy and mounted with a chloral hydrate solution (10 g chloral hydrate, 2.5 ml glycerol, 5 ml water). Slides were kept at room temperature for at least 24 h for clarification. Slices were observed with a differential interference contrast microscope (BX50WI, Olympus), and images were captured with an equipped CCD camera.

## Supplementary information

Supplementary Figures and tables
