# Supplementary material for: Abnormal leaf development of rpt5a mutant under zinc deficiency reveals important role of DNA damage alleviation for normal leaf development
Source: Sci Rep. 2019 Jun 27;9:9369. doi: 10.1038/s41598-019-44789-w (PMC6597565; doi:10.1038/s41598-019-44789-w)
Supplement: Supplementary file 1 — Supplementary Figures and tables [file 41598_2019_44789_MOESM1_ESM.pdf]

Supplementary Information

Title: Abnormal leaf development of *rpt5a* mutant under zinc deficiency reveals important role of DNA damage alleviation for normal leaf development

Naoyuki Sotta[1,†], Takuya Sakamoto[2,†], Sachihiro Matsunaga[2], Toru Fujiwara[1,*]

[1] Department of Applied Biological Chemistry, Graduate School of Agricultural and Life Sciences, The University of Tokyo, 1-1-1 Yayoi, Bunkyo, Tokyo 113-8657, Japan
[2] Department of Applied Biological Science, Faculty of Science and Technology, Tokyo University of Science, 2641 Yamazaki, Noda, Chiba 278-8510, Japan
† These authors equally contributed to this work.
* Correspondence: atoruf@mail.ecc.u-tokyo.ac.jp; Tel.: +81-3-5841-5104

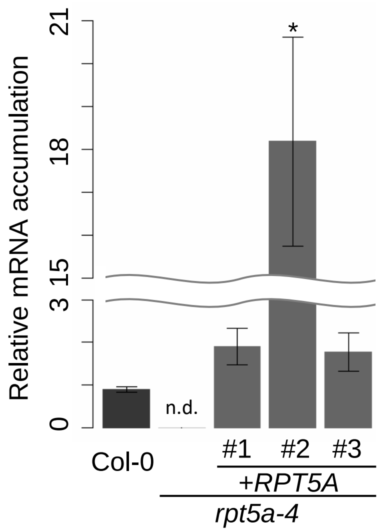

Supplementary Figure 1. *RPT5A* mRNA accumulation in *rpt5a-4* complementation lines. Seedlings were grown for 1 month under normal conditions and total RNA was extracted from the second or third pair of true leaves. *RPT5A* mRNA accumulations were normalized by geometric mean of *Actin8*, *PEX4* and *SAND*. Values represent mean ± standard deviation of 3 biological replicates. The numbers with "#" are identifiers for independent transformant lines in the complementation experiment. An asterisk represents significant difference from Col-0 at $p < 0.05$ by Dunnett's test. n.d., not detected, less than $<9.8 \times 10^{-4}$.

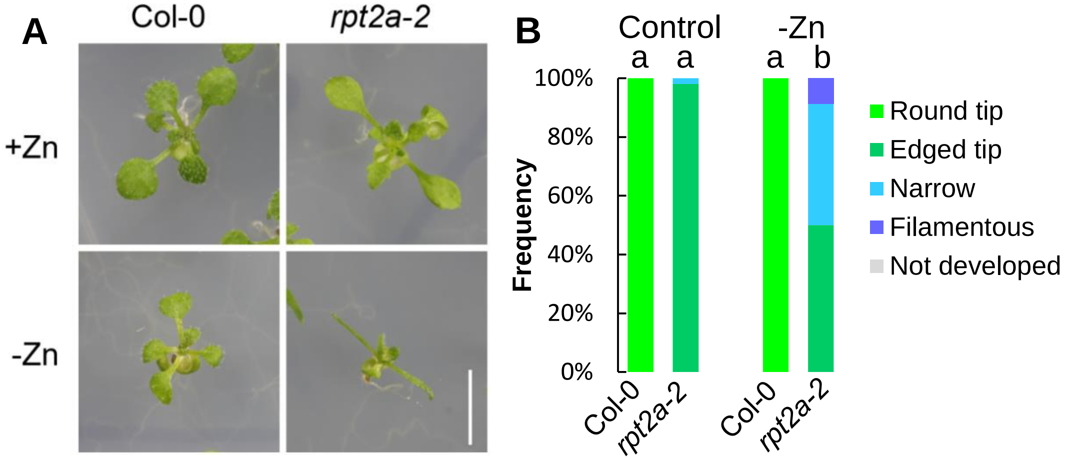

Supplementary Figure 2. Leaf shape of *rpt2a-2* under zinc deficiency. (A) Fifteen-day-old seedlings of the wild type and *rpt2a-1* grown under the control (normal MGRL medium) and excess boron conditions. Bar; 5 mm. (**B**) Leaf shapes were categorized into 4 groups; round tip, edged tip, narrow and filamentous. The typical leaf shapes for each category is illustrated in Figure 1. Frequency was calculated from observation of at least 20 individual plants for each condition. There is no significant difference in the ratio of ("round tip" + "edged tip") : ("narrow" + filamentous") at $p<0.05$ among groups sharing the same alphabet by Fisher's exact test with Holm correction.

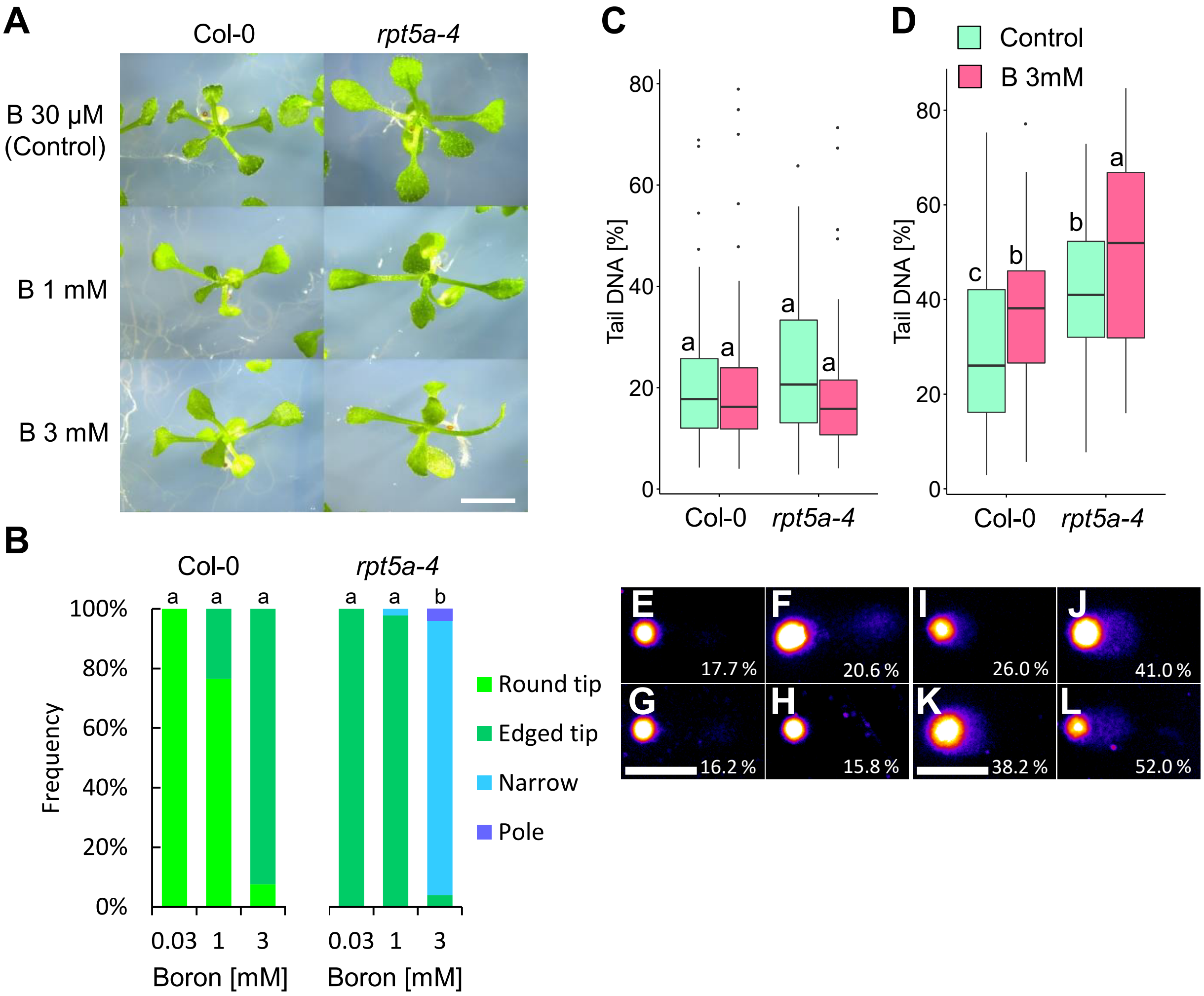

Supplementary Figure 3. Leaf shape and DNA damage accumulation of *rpt5a-4* under excess boron stress. (A) Seventeen-day-old seedlings of the wild type and *rpt5a-4* grown under the control (normal MGRL medium) and excess boron conditions. Bar; 5 mm. (**B**) Leaf shapes were categorized into 4 groups; round tip, edged tip, narrow and filamentous. The typical leaf shapes for each category is illustrated in Figure 1. Frequency was calculated from observation of at least 20 individual plants for each condition. There is no significant difference in the ratio of (“Round tip” + “Edged tip”) : (“Narrow” + “Filamentous”) at $p<0.05$ among groups sharing the same alphabet by Fisher’s exact test with Holm correction. (**C-L**) DSB and SSB accumulations in shoot of *rpt5a-4* under excess boron. Nuclei were extracted from whole shoots of 14-day-old seedlings and were subjected to a comet assay. (**C**) N/N comet assay to detect DSB. (**D**) A/N comet assay to detect DSB and SSB. 66 nuclei were observed for each treatment. Median and first and third quartiles are shown in cross bars and vertical bars represent range of data points obtained. There is no significant difference at $p < 0.05$ between groups sharing the same letter by Steel–Dwass test. (**E–L**) Representative nuclei images from comet assay with N/N method (E-H) and A/N method (I-L). Nuclei with median Tail DNA [%] are shown with their values. (E,I) Wild type, control, (F,J) wild type, 3 mM boric acid, (G,K) *rpt5a-4*, control, (H,L) *rpt5a-4*, 3 mM boric acid. Bars, 50 μm.

Supplementary Table 1. Primers used for real-time PCR.
* AT4G02390 is referred to as PARP1 in some reports but in this report it is referred as to PARP2 based on homology to human genes.

Target gene	Primer name	Sequence	Reference
Actin8	Actin8_RT_F	GCCAGATCTTCATCGTCGTG	
	Actin8_RT_R	TCTCCAGCGAATCCAACCTT	
PEX4 (UBC)	UBC_RT_F	CTGCGACTCAGGGAATCTTCTAA	1
	UBC_RT_R	TTGTGCCATTGAATTGAACCC	1
SAND	SAND_RT_F	AACTCTATGCAGCATTTGATCCACT	1
	SAND_RT_R	TGATTGCATATCTTTATCGCCATC	1
BRCA1	BRCA1_RT_F	CCATGTATTTTGCAATGCGTG	
	BRCA1_RT_R	TGTGGAGCACCTCGAATCTCT	
RAD51	RAD51_RT_F	CGAGGAAGGATCTCTTGCAG	
	RAD51_RT_R	GCACTAGTGAACCCCAGAGG	
PARP2* (AT4G02390)	PARP1_RT_F	GCTTTGGGAGACATGAATGAAC	2
	PARP1_RT_R	AAGTGGAACAACAACACCGTCT	2
AS2	AS2_RT_F	AATTTACTCGCAGGGAGAGG	
	AS2_RT_R	CGCCGGAGGAATTTGCAAGC	
PHABULOSA	PHB_RT_F	TTGGTTTCAGAACCGCAGA	3
	PHB_RT_R	CTGTTTGAAGACGAGCAGCTT	3
PHAVOLUTA	PHV_RT_F	TTGGTTCCAGAATCGCAGA	3
	PHV_RT_R	CACTGTCTGAAGACGAGCTGA	3
ETTTIN	ETTIN-F	CGCCTACTCAATAACCGATCATC	4
	ETTIN-R	ACGGCCCACACCAAATGTT	4
YABBY5	YAB5-F1	ACGCCCTAATTTCCAGGCAAC	4
	YAB5-R1	GTTGCTCAGTTATGGTACGAG	4
RPT5A	RPT5a_RT_F	AAGTCAGCGGAGACAGGGAA	
	RPT5a_RT_R	GCTGCAATCACCTTAATACG	

Supplementary Table 2. Primers used for cloning. The underlined sequence is a required linker for D-TOPO cloning.

Target gene	Primer name	Sequence
RPT5A	RPT5A_F	<u>CACC</u>CTCTAGAGGTTCCCAATTAG
	RPT5A_R	GTGAAGACGAAGGAGTCGAT

References

1. Czechowski, T., Stitt, M., Altmann, T., Udvardi, M. K. & Scheible, W. R. Genome-wide identification and testing of superior reference genes for transcript normalization in *Arabidopsis*. *Plant Physiol.* **139**, 5-17 (2005).
2. Ricaud, L. *et al*. ATM-mediated transcriptional and developmental responses to γ-rays in Arabidopsis. *PloS one* **2**, e430 (2007).
3. Turchi, L. *et al*. Arabidopsis HD-Zip II transcription factors control apical embryo development and meristem function. *Development* **140**, 2118-2129 (2013).
4. Iwasaki, M. *et al*. Dual regulation of ETTIN (ARF3) gene expression by AS1-AS2, which maintains the DNA methylation level, is involved in stabilization of leaf adaxial-abaxial partitioning in Arabidopsis. *Development* **140**, 1958-1969 (2013).
